# Exploration and Improvement of Acid Hydrolysis Conditions for Inulin-Type Fructans Monosaccharide Composition Analysis: Monosaccharide Recovery and By-Product Identification

**DOI:** 10.3390/foods13081241

**Published:** 2024-04-18

**Authors:** Xinyan Zong, Ningyu Lei, Junyi Yin, Weiwei He, Shaoping Nie, Mingyong Xie

**Affiliations:** State Key Laboratory of Food Science and Resources, China-Canada Joint Laboratory of Food Science and Technology (Nanchang), Key Laboratory of Bioactive Polysaccharides of Jiangxi Province, Nanchang University, 235 Nanjing East Road, Nanchang 330047, China; ncuskzongxinyan@163.com (X.Z.); nie68@sina.com (S.N.); xmync@163.com (M.X.)

**Keywords:** inulin-type fructan, hydrolysis condition, monosaccharide composition, hydrolysis by-product

## Abstract

Acid hydrolysis serves as the primary method for determining the monosaccharide composition of polysaccharides. However, inappropriate acid hydrolysis conditions may catalyze the breakdown of monosaccharides such as fructans (Fru), generating non-sugar by-products that affect the accuracy of monosaccharide composition analysis. In this study, we determined the monosaccharide recovery rate and non-sugar by-product formation of inulin-type fructan (ITF) and Fru under varied acid hydrolysis conditions using HPAEC-PAD and UPLC-Triple-TOF/MS, respectively. The results revealed significant variations in the recovery rate of Fru within ITF under different hydrolysis conditions, while glucose remained relatively stable. Optimal hydrolysis conditions for achieving a relatively high monosaccharide recovery rate for ITF entailed 80 °C, 2 h, and 1 M sulfuric acid. Furthermore, we validated the stability of Fru during acid hydrolysis. The results indicated that Fru experienced significant degradation with an increasing temperature and acid concentration, with a pronounced decrease observed when the temperature exceeds 100 °C or the H_2_SO_4_ concentration surpasses 2 M. Finally, three common by-products associated with Fru degradation, namely 5-hydroxymethyl-2-furaldehyde, 5-methyl-2-furaldehyde, and furfural, were identified in both Fru and ITF hydrolysis processes. These findings revealed that the degradation of Fru under acidic conditions was a vital factor leading to inaccuracies in determining the Fru content during ITF monosaccharide analysis.

## 1. Introduction

Fructans, initially discovered by the German scientist Rose in 1804, are fructofuranose-based compounds primarily composed of fructose linkages. They were first isolated from *Inula helenium* L., a plant belonging to the *Asteraceae* family [1]. Plant-derived fructans, due to their isolation from *Inula helenium* and naming as inulin, are also termed as inulin-type fructans (ITF). IFT predominantly consists of β-1,2-linked fructofuranose units. In recent years, numerous investigations delve into a multitude of aspects concerning of ITF, including encompassing extraction, purification, physicochemical properties, structural characteristics, and physiological activities [2,3]. ITF derived from natural plants exhibits a wide range of bioactive functions and demonstrates considerable edible and medicinal value [4]. Furthermore, research on ITF has leveraged the inherent properties of fructans to drive advancements in food technology, health products, and industrial production [5,6].

The determination of monosaccharide composition plays a pivotal role in elucidating the primary structure of polysaccharides [7]. Furthermore, exploring acid hydrolysis conditions for monosaccharide composition analysis is not only necessary for the structural elucidation of ITF but also holds substantial importance for the identification of polysaccharides sourced from diverse origins, types, or compositions. Acid hydrolysis stands as the cornerstone pretreatment method for polysaccharide samples, facilitating the liberation of constituent monosaccharides for subsequent analysis, often employing the high-performance anion exchange chromatography with pulsed amperometric detection (HPAEC-PAD) method. Key variables such as acid hydrolysis duration, temperature, and acid concentration are meticulously adjusted to ensure the optimal recovery, precision, and reproducibility of monosaccharide data [8]. Tailoring hydrolysis conditions to suit the dissociation of polysaccharides with diverse structural characteristics is crucial to achieving accurate results [9,10]. For instance, distinct ketoses (e.g., D-fructose) and aldoses (e.g., D-glucose) may yield identical alditol products upon reduction, owing to the enolization and dehydration processes converting D-fructose to alditol under harsh hydrolytic conditions. Consequently, milder hydrolysis conditions are preferred for samples rich in fructose [11,12,13].

ITF is a furanose fructan characterized but exhibits lower stability in acid hydrolysis compared to pyranose fructans, with a recovery rate of only 80~90% [14,15]. Therefore, when delving into the analysis of ITF’s monosaccharide composition, researchers are inevitably confronted with the challenge posed by its diminished stability during acid hydrolysis. The accuracy of determining its monosaccharide composition is intricately tied to various factors, including monosaccharide variability, their linkages, and configurations. For instance, in the investigation of ITF extracted from *Polygonatum sibiricum*, different hydrolysis temperatures exerted notable effects on the structural characterization efforts. Moreover, under strong acid or high-temperature conditions, carbohydrates can undergo enolization and dehydration reactions, leading to the generation of various non-sugar by-products [16]. For instance, in the acid-catalyzed hydrolysis of ITF, the depolymerization of polysaccharides into free Fru may lead to additional hydrolysis, resulting in the formation of certain furfural by-products [17,18]. This process compromises the accuracy and reliability of experimental data, posing challenges to the structural characterization of ITF [19,20].

Additionally, the process yields isomaltol, hydroxyacetylfuran carboxylic acid, formic acid, acetic acid, and propionic acid [21,22]. Inappropriate hydrolysis methodologies result in the degradation of polysaccharides into non-sugar compounds, leading to incorrect estimations of monosaccharide content in ITF. Consequently, proper modification of experimental hydrolysis conditions, considering the unique properties inherent to diverse polysaccharides, constitutes an imperative consideration [23]. The customization and optimization of acid hydrolysis conditions tailored to the specific attributes of a given polysaccharide are pivotal for ensuring accuracy in the analysis of its monosaccharide composition and facilitating structural characterization [24,25].

In this study, we explored optimal acid hydrolysis conditions and analyzed hydrolysis by-products of ITF in varying acid concentrations, hydrolysis time, and temperatures. Using the HPAEC-PAD method to determine the monosaccharide recovery rates of ITF under various acid hydrolysis conditions, we optimized the acid hydrolysis conditions for IFT monosaccharide composition analysis. Furthermore, the impact of acid hydrolysis conditions on Fru recovery rates was validated through Fru standard samples. Subsequently, in order to explore the main reasons for the inaccurate determination of fructose content in the analysis of ITF monosaccharide composition, we identified the hydrolysis by-products in IFT and fructose standard samples using UPLC-Triple-TOF/MS. Based on this study, we aim to provide theoretical basis for the hydrolysis and structural analysis of ITF.

## 2. Materials and Methods

### 2.1. Materials and Reagents

Inulin-type fructan was obtained from BENEO-Orafti (Oreye, Belgium). Monosaccharide standards, D-glucose (Glc) and D-fructose (Fru), were purchased from Sigma-Aldrich (Bratislava, Slovakia). Oasis HLB DISKS were acquired from Waters Corporation (Milford, MA, USA). Sulfuric acid (H_2_SO_4_, GR) was purchased from Sinopharm Chemical Reagent Co., Ltd. (Shanghai, China). All other chemicals used in this study were of analytical grade and obtained commercially. 

### 2.2. Hydrolysis and Monosaccharide Composition Analysis

To explore the stability of monosaccharides under different hydrolysis conditions [26], the Fru and ITF were hydrolyzed under the same conditions. The samples (5 mg each) were individually dissolved in 0.5 mL of H_2_SO_4_ and stirred for 30 min in an ice bath. Hydrolysis reactions were subsequently conducted under varied conditions, including different temperatures (40, 60, 80, 100, and 120 °C), acid concentrations (0.5, 1 and 2 M), and heating durations (0.25, 0.5, 1, and 2 h). When the reaction was completed, the sample was diluted to a volume of 20 μg/mL before chromatography analysis.

The monosaccharide composition was analyzed by HPAEC (Dionex ICS-6000 system, Thermo Fisher Scientific, Waltham, MA, USA), and pulsed amperometric detector (PAD) equipped with working electrode (gold electrode) and reference electrode (Ag/AgCl) was used for detection. Dionex Carbo PacTM PA-20 (3 mm × 30 mm) guard column and PA-20 (3 mm × 150 mm) analytical column was adopted for separation [13]. Gradient elution was performed using 250 mmol/L NaOH solution, deionized water, and 1 mol/L NaOAc solution as mobile phases at a flow rate of 0.5 mL/min. The column and detector temperatures were 30 °C and 25 °C, respectively, while the injection volume was 10 μL. The detection was carried out using a sugar standard four potential method. The monosaccharide standards and these samples were processed and analyzed in parallel.

### 2.3. Desalination and Hydrolysis By-Products Identification 

To identify the hydrolysis by-products under different hydrolysis conditions, the experiment was divided into three groups with significant differences based on the hydrolysis conditions set in 0: incomplete acid hydrolysis conditions (IN: hydrolyze with 0.5 M H_2_SO_4_ at 40 °C for 0.5 h), better acid hydrolysis conditions (BE: hydrolyze with 1 M H_2_SO_4_ at 80 °C for 2 h), and excessive acid hydrolysis conditions (EX: hydrolyze with 1 M H_2_SO_4_ at 120 °C for 1 h).

After hydrolysis, the sodium hydroxide solution was used to neutralize the residual H_2_SO_4_ in the hydrolyzed solution, maintaining the pH of the reaction system at 7.0. Solid phase extraction was carried out using a vacuum solid phase extraction device and an activated Waters Oasis HLB cartridge (200 mg, 6 cc) solid phase extraction column to extract and desalt the neutralized sample. The sample was washed through the extraction column with 2.5 mL of deionized water, followed by washing and collecting the eluent with 1.5 mL of methanol solution. Finally, the eluent was collected and passed through a 0.22 μm organic filter and transferred to a sample vial for subsequent UPLC-Triple-TOF/MS determination and analysis [27,28]. Three parallel eluents were prepared for each sample. 

UPLC-Triple-TOF/MS was employed to identify compounds generated in the reaction systems under different hydrolysis conditions and analyze the association between hydrolysis conditions and side reactions based on changes in characteristic differential compounds. The chromatographic column used was an ACQUITY UPLC HSS T3 (100 Å, 50 mm × 2.1 mm, 1.8 μm). The mobile phases were 0.1% formic acid aqueous solution and methanol at a flow rate of 0.25 mL/min. The injection volume was 5 μL. Mass spectrometric detection was performed in positive/negative ionization modes.

### 2.4. Statistical Analysis

The monosaccharide standards at different concentrations were analyzed by HPAEC-PAD to establish standard curves with peak area integration as the variable. This was used to calculate the monosaccharide recovery rates of the aforementioned samples. Data plotting was performed using Origin Pro 2017C (version B9.4.2.380, Stat-Ease Inc., Minneapolis, MN, USA). The results are expressed as mean ± standard deviation (SD). 

The hydrolysis products of Fru and ITF under three different acidic hydrolysis conditions were determined by UPLC-Triple-TOF/MS. Feature compounds were preprocessed using Progenesis QI software (V 2.1 ASMS 2015). After preprocessing, the data were uploaded to MetaboAnalyst 5.0 (www.metaboanalyst.ca, accessed on 8 September 2023) [28,29] for chemometric analysis. The chemical categories of compounds were queried on the PubChem website (https://pubchem.ncbi.nlm.nih.gov/, accessed on 18 September 2023). 

## 3. Results and Discussion

### 3.1. Optimization of Acid Hydrolysis Conditions of ITF

**Figure 1:**
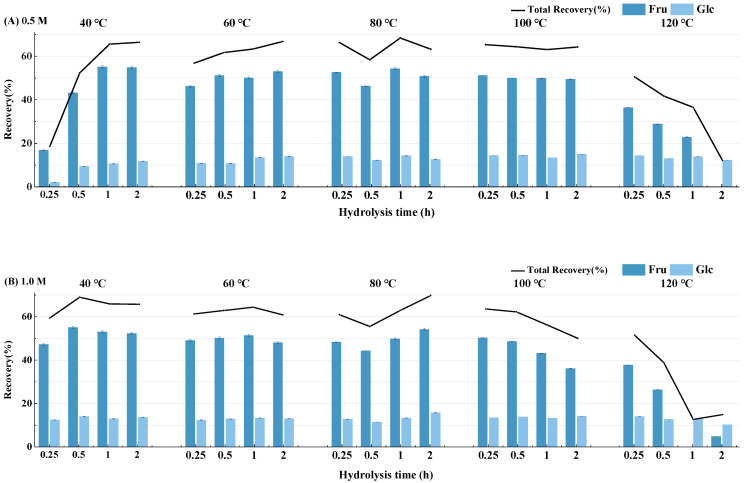

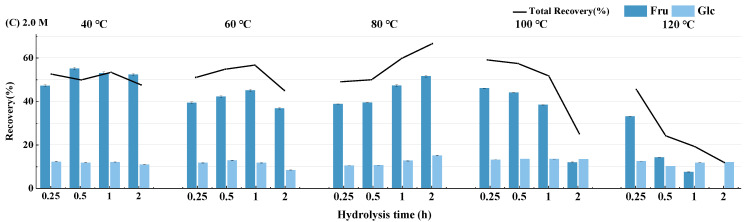
The fluctuation of monosaccharide (Fru and Glc) recovery rates (%) and total recovery rates (%) over time during the hydrolysis of inulin-type fructans (ITF) under different temperature conditions (ranging from 40 °C to 120 °C) at (**A**) 0.5 M, (**B**) 1.0 M, and (**C**) 2.0 M H_2_SO_4_ concentrations.

Previous studies have shown that ITF undergoes hydrolysis into Glc and Fru in H_2_SO_4_ solution. However, Fru may exhibit poor stability under acidic hydrolysis conditions, potentially resulting in inaccuracies in the determination of monosaccharide composition. To investigate the changes in monosaccharide recovery rate of ITF under varying hydrolysis conditions and determine the optimal hydrolysis conditions, ITF was hydrolyzed at different conditions, including H_2_SO_4_ concentrations of 0.5 M, 1 M, and 2 M, temperatures ranging from 40 °C to 120 °C, and reaction times of 0.25 h, 0.5 h, 1 h, and 2 h. The relative percentage of the recovery rate from each experiment is shown in Figure 1.

In general, the recovery rates of these two monosaccharides varied under different hydrolysis conditions. However, compared to the recovery rate of Fru, the recovery rate of glucose from ITF remained relatively stable under different conditions. Specifically, under relatively mild acid condition (0.5 M H_2_SO_4_) and temperature rang (from 40 °C to 60 °C), the recovery rate of Fru increased with reaction time. However, when the hydrolysis temperature exceeded 100 °C and the concentration of H_2_SO_4_ exceeded 1 M, the Fru recovery rate experienced a dramatic decrease. Therefore, this hydrolysis condition was optimal for the determination of monosaccharides in ITF. In addition, these results indicated that Fru can achieve a relatively ideal range of the hydrolysis recovery rates under mild hydrolysis conditions [30]. Once the hydrolysis conditions meet the requirements for monosaccharide release, the variation in the recovery rate is no longer dependent on whether the hydrolysis intensity is sufficient to support monosaccharide release but rather on the stability of the monosaccharide in the hydrolytic environment [27,31]. However, when hydrolysis conditions (e.g., acid strength or temperature) exceed the optimal parameters, with increasing hydrolysis intensity, Fru may gradually degrade and convert into non-sugar substances, resulting in a significant decrease in the recovery rate, approaching zero [27]. 

In contrast to Fru, the Glc released from ITF exhibited stable hydrolysis recovery rates under the different hydrolysis conditions set in the experiment. As shown in Figure 1, during the initial hydrolysis process at 40 °C using 0.5 M sulfuric acid hydrolysis for 0.25~1 h and 1 M sulfuric acid hydrolysis for 0.25~0.5 h, the recovery rate of Glc gradually increased, indicating the gradual release of free Glc monosaccharides from ITF, reaching a recovery rate of approximately 15%. Subsequently, even with the increased hydrolysis intensity up to 120 °C with 2 M sulfuric acid for 2 h, no significant trend of Glc loss or degradation was observed, and the recovery rate remained relatively stable within the range of 10% to 16%. These results indicated that Glc may not achieve satisfactory recovery rates under low hydrolysis intensities but exhibits good stability under subsequent severe hydrolysis conditions, with relatively minor variations influenced by the different hydrolysis conditions within the specified range [32,33]. 

Therefore, under the conditions of 1 M H_2_SO_4_ at 80 °C for 2 h, both fructose and glucose exhibit better recovery rates, making it the optimal hydrolysis condition for determining the composition of ITF monosaccharides (Figure 1). However, taking into account practical considerations and efficiency, it can be inferred that a broader range of acid hydrolysis conditions, spanning temperatures from 60 to 100 °C, sulfuric acid concentrations ranging from 0.5 to 1 M, and hydrolysis duration of 0.5 to 2 h, could be deemed as suitable for the hydrolysis of ITF. Subsequently, we employed the optimized acid hydrolysis conditions (1 M sulfuric acid hydrolysis for 2 h at 100 °C) to elucidate the structural characteristics of ITF from *Polygonatum sibiricum* [34,35].

### 3.2. Stability of Monosaccharide Standards under Different Hydrolysis Conditions 

**Figure 2:**
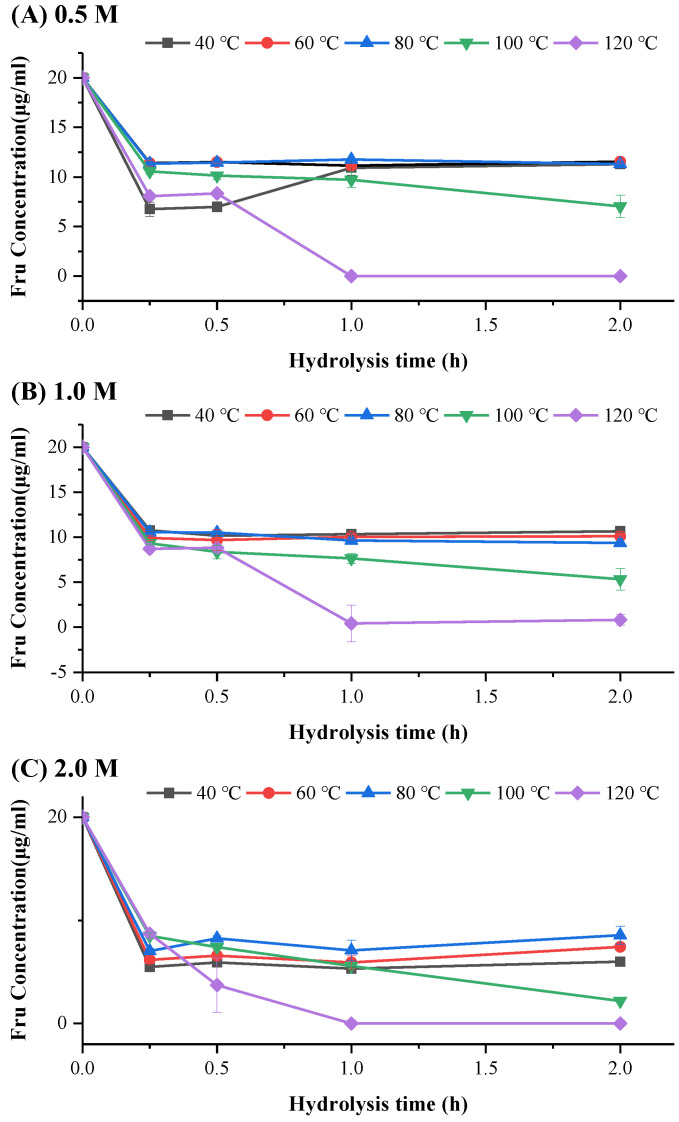
The fluctuation in fructose (Fru) standard monosaccharide concentration over hydrolysis time under different temperature conditions (ranging from 40 °C to 120 °C), while H_2_SO_4_ concentrations were (**A**) 0.5 M, (**B**) 1.0 M, and (**C**) 2.0 M.

Previous studies suggest that Fru exhibits poorer stability compared to other monosaccharide such as glucose or galactose [22]. During acid hydrolysis, monosaccharide (e.g., Fru) released from the polysaccharide chain may undergo further degradation to produce by-products such as 5-hydroxymethyl-2-furaldehyde, hydroxyacetylfuran, 5-methyl-2-furaldehyde, hydroxyacetylfuran formic acid, formic acid, acetic acid, and levulinic acid [21,36]. The degradation rate and degree of fructose increase with higher levels of acid concentration, longer reaction time, and elevated temperature [27,31]. To elucidate how these hydrolysis conditions affect the content and stability of free fructose released from ITF, we examined the acid stability of fructose. Figure 2 shows the fluctuation in Fru standard monosaccharide concentration over hydrolysis time under different temperature and H_2_SO_4_ concentrations. The recovery rate obtained from each experiment is expressed in relative percentage and listed in Supplementary Data Table S1.

The results showed that Fru underwent significant degradation with the progression of hydrolysis under various conditions, resulting in a decrease in concentration, and generally stabilizes after 1 h (Figure 2). Furthermore, with increasing hydrolysis temperature and H_2_SO_4_ concentrations, the Fru concentration exhibited a decrease trends. Specifically, when the concentration of H_2_SO_4_ reached 2 M or the temperature exceeded 100 °C, the Fru recovery rate significantly decreased or even approaching 0. This is in accordance with several previous studies [31,37], supporting the hypothesis that excessively strong hydrolysis conditions during the hydrolysis of ITF can lead to fructose degradation, thereby reducing the accuracy of Fru composition results. 

### 3.3. Analysis of Hydrolysis By-Products 

#### 3.3.1. Trend of Hydrolysis By-Products of ITF under Different Hydrolysis Conditions

The hydrolysis reaction of polysaccharides is a complex process that involves not only the breakdown of polysaccharides into oligosaccharides and monosaccharides but also various side reactions leading to the formation of non-sugar compounds such as furfural [22,28]. To investigate the types and variations in the content of non-sugar compounds generated during the hydrolysis process of ITF, UPLC-Triple-TOF/MS analysis was employed to determine the relevant compound profiles under different acid hydrolysis conditions, including incomplete hydrolysis conditions (IN) with 0.5 M H_2_SO_4_ at 40 °C for 0.5 h, better hydrolysis conditions (BE) with 1 M H_2_SO_4_ at 80 °C for 2 h, and excessive hydrolysis conditions (EX) with 1 M H_2_SO_4_ at 120 °C for 1 h. Furthermore, employing Progenesis QI software for data processing and statistical analysis, a total of 585 characterized compounds were selected from 7456 and 5179 original data in positive and negative ion modes from Fru and ITF hydrolysis solution.

**Figure 3:**
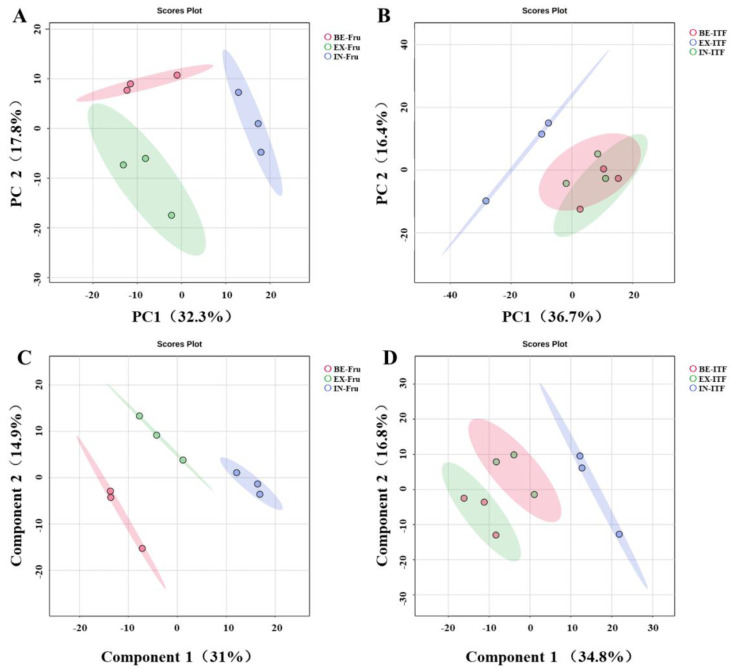
PCA score plots and PLS-DA score plots in three acidic hydrolysis conditions. (**A**) PCA score plots of Fru; (**B**) PCA score plots of ITF; (**C**) PLS-DA score plots of Fru; (**D**) PLS-DA score plots of ITF.

Based on the identification of compounds obtained in three different hydrolysis conditions (the three hydrolysis conditions group IN, BE, and EX are introduced in 0), chemometric analysis methods were performed to investigate the differential features of Fru and ITF. Further clarification of the differences in compound generation under different hydrolysis conditions was carried out by using principal component analysis (PCA) and partial least squares discriminant analysis (PLS-DA) score plots, as shown in Figure 3. The results indicated a certain degree of dispersion among the different groups, suggesting the presence of differences in the compounds generated under different hydrolysis conditions.

**Figure 4:**
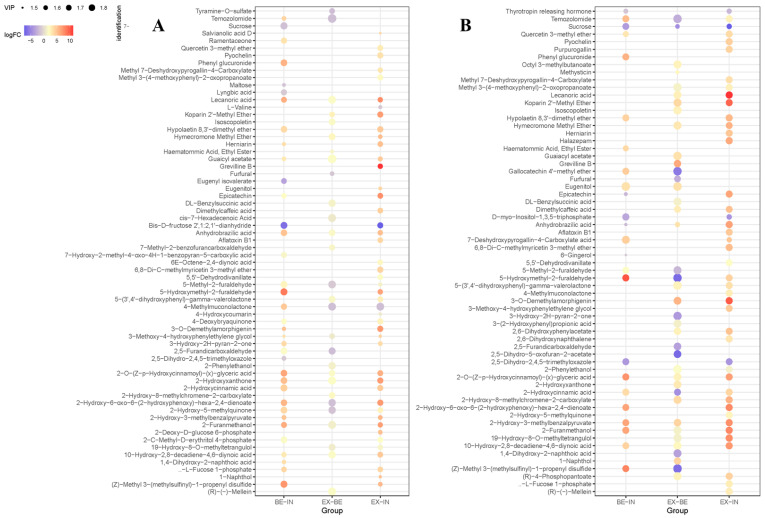
Dot map of differentiated compounds from Fru (**A**) and ITF (**B**) under different hydrolysis conditions. The color and size of the bubbles represent the Log_2_FC and VIP values, respectively. IN: incomplete acid hydrolysis conditions (0.5 M H_2_SO_4,_ 40 °C, 0.5 h); BE: better acid hydrolysis conditions (1 M H_2_SO_4_, 80 °C, 2 h); EX: excessive acid hydrolysis conditions (1 M H_2_SO_4,_ 120 °C, 1 h).

Figure 4A and Figure 4B, respectively, display comparative analyses of the hydrolysis by-products of Fru and ITF under varying acid intensities. In general, numerous by-products resulting from the hydrolysis of Fru and ITF underwent significant alterations as the hydrolysis conditions intensified, which is consistent with previous reports [38]. Furthermore, previous studies have documented that the generation of by-products resulting from ITF hydrolysis primarily comprising furfural compounds [36,39,40]. 

During Fru hydrolysis and ITF hydrolysis, the levels of furfural compounds, such as 5-hydroxymethyl-2-furaldehyde (5-HMF) and 5-methyl-2-furaldehyde (5-MF), varied among different hydrolysis conditions. During Fru hydrolysis, the content of 5-HMF accumulated gradually with increased hydrolysis degree, while 5-MF only exhibited a slight increase in the EX and BE groups compared to the IN group. Interestingly, furfural (FF) exhibited a decrease in the EX group compared to the BE group during Fru hydrolysis. During ITF hydrolysis, both 5-HMF and 5-MF underwent a trend of initially rising and then declining as the acid hydrolysis conditions intensified (IN → BE → EX), while furfural (FF) shows a lower concentration in the EX group compared to the BE group. Due to the intensification of hydrolysis conditions, both in the hydrolysis of Fru and ITF, there was a significant reduction in Fru in the products. Therefore, it can be inferred that intensified hydrolysis conditions may lead to the instability of the furfural compound generated from Fru conversion, resulting in its further decomposition or conversion into various by-products, including 2-hydroxyacetofuran [41], 5-chloromethylfurfural [42], furan formaldehyde, acetylpropionic acid, formic acid, etc. For instance, according to a report by Asghari FS and Yoshida H (2006), it has been observed that at lower pH levels, 5-HMF may convert into levulinic and formic acids [43].

#### 3.3.2. Identification of Characteristic Hydrolysis By-Product

To investigate the similarity between the main by-products generated during the hydrolysis of fructose and ITF, we compiled a list of common by-products potentially presented in the hydrolysis products of both Fru and ITF, totaling 57 types, as shown in Table S2. Among these, the compounds potentially linked to sugar degradation comprise the 13 substances listed in Table 1. The presence of furfural, acids, alcohols, and aldehydes in the sample corroborates with the reported literature on sugar degradation products [44,45,46].

**Figure 5:**
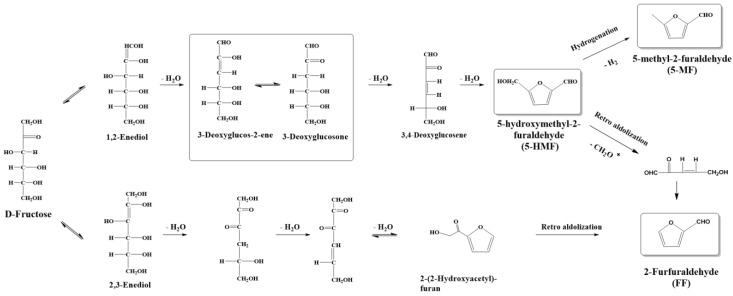
Pathways related to the formation of furfural from fructose.

The three common degradation products of Fru, 5-hydroxymethyl-2-furaldehyde, 5-methyl-2-furaldehyde, and furfural, were detected in the products of both Fru and ITF under different hydrolysis conditions, indicating that Fru generated by ITF hydrolysis also underwent degradation through pathways associated with 5-HMF [36]. As illustrated in Figure 5, previous studies have shown that under acidic conditions, Fru can undergo degradation through two pathways, leading to the formation of furfural compounds. This study unveiled notable fluctuations in 5-HMF and 5-MF levels with alterations in hydrolysis conditions (Figure 4), suggesting that, under the current hydrolysis parameters, Fru predominantly degrades through pathways associated with 5-HMF [47,48].

Hence, there is a critical need to concentrate more on methods to limit the conversion of Fru to 5-HMF during the hydrolysis of ITF to enhance the accuracy of fructose content determination in ITF.

**Table 1:** The common differentiated sugar degradation-associated compounds of Fru and ITF under three acid hydrolysis conditions.

No	Identification	*m*/*z* ^a^	Adducts	Error (ppm)	RT ^b^ (min)	Score	Formula	Classification ^c^	References
1	2,5-Furandicarboxaldehyde	125.02	[M+H]^+^	−3.493	3.806	46.1	C_6_H_4_O_3_	Aldehyde	[16,44,46]
2	5-Hydroxymethyl-2-furaldehyde	109.0285	[M+H−H_2_O]^+^	0.419	3.660	43	C_6_H_6_O_3_		
3	Furfural	97.03	[M+H]^+^	−2.110	3.101	40.4	C_5_H_4_O_2_		
4	5-Methyl-2-furaldehyde	111.0438	[M+H]^+^	−4.408	4.007	38.7	C_6_H_6_O_2_		
5	2-Phenylethanol	123.0799	[M+H]^+^	−2.350	5.077	37.3	C_8_H_10_O		
6	2-Hydroxycinnamic acid	165.05	[M+H]^+^	−0.838	3.540	41.6	C_9_H_8_O_3_	Acid	[44,49,50]
7	1,4-Dihydroxy-2-naphthoic acid	205.05	[M+H]^+^	−2.377	4.034	38.3	C_11_H_8_O_4_		
8	2-Hydroxy-8-methylchromene-2-carboxylate	207.06	[M+H]^+^	−2.350	5.077	38.9	C_11_H_10_O_4_	Ester	[51,52]
9	2-Hydroxy-3-methylbenzalpyruvate	207.06	[M+H]^+^	−1.987	5.699	41.1	C_11_H_10_O_4_	Ketone	[45,50]
10	2-Hydroxy-5-methylquinone	139.04	[M+H]^+^	−1.921	3.819	39.7	C_7_H_6_O_3_	Others	[38,51,53]
11	2-Hydroxy-6-oxo-6-(2-hydroxyphenoxy)-hexa-2,4-dienoate	273.04	[M+H]^+^, [M+Na]^+^, [M+H−H_2_O]^+^	−0.387	4.209	39.7	C_12_H_10_O_6_		
12	(R)-(−)-Mellein	179.07	[M+H]^+^	−1.985	4.847	38.6	C_10_H_10_O_3_		
13	2-Hydroxyxanthone	213.0541	[M+H]^+^	−2.367	6.092	38.5	C_13_H_8_O_3_		

Note: ^a^, experimental data; ^b^. Retention time; ^c^. Classification, based on Pubmed website query results.

## 4. Conclusions

In this study, the optimal hydrolysis conditions for monosaccharide compositional of ITF were explored, and the results revealed that Fru was more susceptible to degradation compared to Glc, and milder hydrolysis conditions were more suitable for preserving the integrity of ITF. The optimal hydrolysis conditions for achieving relatively high yields and satisfactory stability of ITF in optimized hydrolysis conditions (80 °C, 2 h, and 1 M sulfuric acid). Furthermore, this study investigated the by-products generated during the acid hydrolysis of Fru and ITF, and the results indicated that, with the intensification of hydrolysis conditions, 5-hydroxymethyl-2-furaldehyde, 5-methyl-2-furaldehyde, and furfural were closely associated with fructose degradation. This indicates that the liberated free fructose during ITF hydrolysis undergoes further degradation through pathways associated with 5-HMF. Based on the above conclusions, this study provides some theoretical foundations for the establishment of appropriate hydrolysis conditions and the reduction in the side reactions in the in-depth physicochemical analysis of polysaccharides. In addition, to enhance our comprehension of Fru acid degradation and its influencing factors, it will be crucial to conduct a quantitative analysis of Fru by-product content in future studies.

## Data Availability

The original contributions presented in the study are included in the article/Supplementary Material, further inquiries can be directed to the corresponding author.

## Supplementary Materials

The following supporting information can be downloaded at: https://www.mdpi.com/article/10.3390/foods13081241/s1, Table S1: Recovery rate (%) of ITF monosaccharides composition under different H_2_SO_4_ concentration, hydrolysis time and temperature (n = 3); Table S2. Identified compounds potentially presented in the hydrolysis products of both fructose (Fru) and isomaltulose (ITF).
