# Supplementary material for: Exploration and Improvement of Acid Hydrolysis Conditions for Inulin-Type Fructans Monosaccharide Composition Analysis: Monosaccharide Recovery and By-Product Identification
Source: Foods. 2024 Apr 18;13(8):1241. doi: 10.3390/foods13081241 (PMC11049111; doi:10.3390/foods13081241)
Supplement: Supplementary file 1 [file foods-13-01241-s001.zip › foods-2932278-supplementary.pdf]

<u>Supplementary data</u>

Optimizing acid hydrolysis for monosaccharide compositional and analysis for hydrolysis by-product of inulin-type Fructan

State Key Laboratory of Food Science and Technology, Nanchang University, 235 Nanjing East Road, Nanchang, Jiangxi Province, 330047, China

*Corresponding author.
Email: weiweihe_fs@ncu.edu.cn

Table S1. Recovery rate (%) of ITF monosaccharides composition under different H_2SO_4 concentration, hydrolysis time and temperature (n=3)

Temperature/℃		40			60			80		
Acidic concentration/M	Time/h	Fru	Glc	Total	Fru	Glc	Total	Fru	Glc	Total
0.5	0.25	16.81±0.17	2.07±0.02	18.88	46.15±0.46	10.86±0.11	57.02	52.62±0.03	13.87±0.01	66.49
	0.5	43.15±0.43	9.39±0.09	52.53	51.14±0.51	10.7±0.11	61.84	46.3±0.01	12.27±0.01	58.57
	1	55.14±0.55	10.61±0.11	65.75	50.01±0.5	13.49±0.13	63.50	54.31±0.54	14.28±0.14	68.59
	2	54.81±0.55	11.76±0.12	66.58	52.97±0.53	13.97±0.14	66.94	50.79±0.51	12.61±0.13	63.40
1	0.25	47.21±0.47	12.46±0.12	59.67	49.1±0.49	12.37±0.12	61.47	48.33±0.07	12.79±0.02	61.12
	0.5	55.11±0.55	14.04±0.14	69.15	50.17±0.5	12.86±0.13	63.03	44.24±0.03	11.43±0.01	55.68
	1	53.03±0.53	13.05±0.13	66.08	51.34±0.51	13.24±0.13	64.58	49.78±0.5	13.3±0.13	63.08
	2	52.34±0.52	13.6±0.14	65.94	48.03±0.48	13.02±0.13	61.05	54.17±0.54	15.78±0.16	69.95
2	0.25	40.39±0.4	12.35±0.12	52.74	39.42±0.39	11.79±0.12	51.22	38.81±0.05	10.43±0.01	49.24
	0.5	38.21±0.38	11.89±0.12	50.11	42.17±0.42	12.85±0.13	55.01	39.47±0.08	10.71±0.03	50.18
	1	41.44±0.41	12.15±0.12	53.58	45.12±0.45	11.79±0.12	56.91	47.31±0.47	12.74±0.13	60.05
	2	36.85±0.37	10.98±0.11	47.84	36.83±0.37	8.44±0.08	45.27	51.63±0.52	15.14±0.15	66.77

(Continued Table)

Temperature/℃		100			120		
**Acidic concentration/M**	**Time/h**	**Fru**	**Glc**	**Total**	**Fru**	**Glc**	**Total**
0.5	0.25	51.17±0.01	14.33±0.01	65.51	36.39±0.01	14.3±0.01	50.69
	0.5	49.98±0.01	14.55±0.01	64.53	28.89±0.02	12.98±0.01	41.87
	1	49.85±0.05	13.39±0	63.24	22.84±0.16	13.84±0.02	36.68
	2	49.43±0.09	14.97±0.01	64.40	-	12.29±0.01	12.29
1	0.25	50.3±0.03	13.43±0	63.73	37.69±0.03	14.01±0.01	51.70
	0.5	48.62±0.03	13.82±0	62.44	26.32±0.09	12.63±0.01	38.95
	1	43.19±0.03	13.24±0	56.43	-	12.88±0.02	12.88
	2	36.09±0.17	14.16±0.01	50.25	4.81±0.03	10.24±0.00	15.05
2	0.25	46.1±0.03	13.19±0.01	59.29	33.13±0.03	12.56±0.01	45.69
	0.5	44.1±0.04	13.54±0	57.63	14.24±0.06	10.16±0.00	24.40
	1	38.43±0.08	13.49±0.01	51.93	7.58±0.05	11.79±0.02	19.37
	2	11.93±0.22	13.43±0	25.36	-	12.09±0.00	12.09

Data format: Mean ± Standard Deviation (SD)
-: Not detected

Table S2. Identified compounds potentially presented in the hydrolysis products of both fructose (Fru) and isomaltulose (ITF).

No	Common degradation products of Fru and ITF
1	(R)-(-)-Mellein
2	(Z)-Methyl 3-(Methylsulfinyl)-1-Propenyl Disulfide
3	1,4-Dihydroxy-2-Naphthoic acid
4	10-Hydroxy-2,8-Decadiene-4,6-diynoic acid
5	19-Hydroxy-8-O-Methyltetrangulol
6	1-Naphthol
7	2,5-Dihydro-2,4,5-Trimethyloxazole
8	2,5-Furandicarboxaldehyde
9	2-C-Methyl-D-Erythritol 4-Phosphate
10	2-Furanmethanol
11	2-Hydroxy-3-Methylbenzalpyruvate
12	2-Hydroxy-5-Methylquinone
13	2-Hydroxy-6-oxo-6-(2-Hydroxyphenoxy)-Hexa-2,4-Dienoate
14	2-Hydroxy-8-Methylchromene-2-carboxylate
15	2-Hydroxycinnamic acid
16	2-Hydroxyxanthone
17	2-O-(Z-p-Hydroxycinnamoyl)-(x)-Glyceric acid
18	2-Phenylethanol
19	3-Hydroxy-2H-Pyran-2-one
20	3-Methoxy-4-Hydroxyphenylethylene Glycol
21	3-O-Demethylamorphigenin
22	4-Hydroxycoumarin
23	4-Methylmuconolactone
24	5-(3',4'-Dihydroxyphenyl)-Gamma-Valerolactone
25	5,5'-Dehydrodivanillate
26	5-Hydroxymethyl-2-Furaldehyde
27	5-Methyl-2-furaldehyde
28	6,8-Di-C-methylmyricetin 3-Methyl Ether
29	6-Methoxymellein

30	7-Methyl-2-Benzofurancarboxaldehyde
31	Anhydrobrazilic acid
32	Calcium Propionate
33	Dimethylcaffeic acid
34	DL-Benzylsuccinic acid
35	Epicatechin
36	Epigallocatechin
37	Eugenitol
38	Furfural
39	Guaiacylacetone
40	Haematommic acid, Ethyl Ester
41	Herniarin
42	Hymecromone Methyl Ether
43	Hypolaetin 8,3'-Dimethyl Ether
44	Isoscopoletin
45	Koparin 2'-Methyl Ether
46	Lecanoric acid
47	Methyl 3-(4-Methoxyphenyl)-2-Oxopropanoate
48	Methyl 7-Deshydroxypyrogallin-4-Carboxylate
49	Phenyl Glucuronide
50	Pyochelin
51	Quercetin 3-Methyl Ether
52	Ramentaceone
53	Ser-Asp-OH
54	Sodium (±)-2-(4-Methoxyphenoxy)Propionate
55	Sucrose
56	Temozolomide
57	β-L-Fucose 1-Phosphate
